# CP-25 Attenuates the Activation of CD4^+^ T Cells Stimulated with Immunoglobulin D in Human

**DOI:** 10.3389/fphar.2018.00004

**Published:** 2018-01-23

**Authors:** Yu-jing Wu, Heng-shi Chen, Wen-sheng Chen, Jin Dong, Xiao-jie Dong, Xing Dai, Qiong Huang, Wei Wei

**Affiliations:** Institute of Clinical Pharmacology, Anhui Medical University, Key Laboratory of Anti-inflammatory and Immune Medicine, Ministry of Education, Anhui Collaborative Innovation Center of Anti-inflammatory and Immune Medicine, Hefei, China

**Keywords:** immunoglobulin D, immunoglobulin D receptor, CP-25 (paeoniflorin-6′-*O*-benzene sulfonate), T cells, Lck

## Abstract

Researchers have shown that the level of immunoglobulin D (IgD) is often elevated in patients with autoimmune diseases. The possible roles of IgD on the function of human T cell activation are still unclear. Paeoniflorin-6′-*O*-benzene sulfonate (code: CP-25), the chemistry structural modifications of paeoniflorin, was a novel drug of anti-inflammation and immunomodulation. The aims of this study were to determine if human CD4^+^ T cells could be activated by IgD via the IgD receptor (IgDR)-Lck pathway and whether the novel compound CP-25 could affect the activation of T cells by regulating Lck. Human CD4^+^ T cells were purified from peripheral blood mononuclear cells using microbeads. T cell viability and proliferation were detected by Cell Counting Kit-8 and CFSE Cell Proliferation Kit. Cytokines secreted by T cells were assessed with the Quantibody Human Inflammation Array. The binding affinity and expression of IgDR on T cells were detected by flow cytometry, and protein expression of IgDR, Lck, and P-Lck were analyzed by western blot. IgD was shown to bind to IgDR on CD4^+^ T cells in a concentration-dependent manner and stimulate the activation and proliferation of these cells by enhancing phosphorylation of the activating tyrosine residue of Lck (Tyr^394^). CP-25 inhibited the IgD-stimulated activation and proliferation of CD4^+^ T cells, as well as the production of inflammatory cytokines; it was thus suggested that this process might be related to the downregulation of Lck (Tyr^394^) phosphorylation. These results demonstrate that IgD amplifies the activation of CD4^+^ T cells, which could be mediated by Lck phosphorylation. Further, CP-25, via its ability to modulate Lck, is a novel potential therapeutic agent for the treatment of human autoimmune diseases.

## Introduction

T cells are critical for vertebrate adaptive immune responses, during which they function as both regulators and effectors of these processes (Oh and Ghosh, 2013). Abnormalities in T cells have frequently been reported in autoimmune diseases such as rheumatoid arthritis (RA), systemic lupus erythematosus (SLE), and multiple sclerosis (MS), which is characterized by exaggerated activation of CD4^+^ T cells (He et al., 2016; van Loosdregt et al., 2016). Thus, it is essential to explore the pathogenesis of abnormally activated T cells in autoimmune diseases for the discovery of new drugs. Immunoglobulin D (IgD) includes membrane IgD (mIgD) and secreted IgD (sIgD), which remains a rather enigmatic player in the immune system. Reports have shown that sIgD might be increased in patients with autoimmune diseases (Koelsch et al., 2007) such as RA and SLE, consistent with our finding of elevated sIgD in RA (Wu et al., 2016). In addition, the IgD receptor (IgDR), which is expressed on T cells, can bind to sIgD to activate downstream signal transduction. Our previous study determined that excessive IgD can result in hyperactive CD4^+^ T cells, mediated by IgDR, which might indicate a common pathogenic mechanism of RA (Wu et al., 2016). Nguyen reported that the administration of anti-IgD can significantly attenuate the progression of experimental arthritis, indicating that IgD participates in CD4^+^ T cell-mediated autoimmune arthritis (Nguyen et al., 2010). These studies suggest that IgD plays a crucial part in the pathology of autoimmune diseases, which might be related to the exaggerated activation of CD4^+^ T cells. Rodent studies showed that the inhibition of protein tyrosine kinases (PTKs) can block the upregulation of IgD-induced IgDR, which was shown to involve Lck, Fyn, ZAP-70, and PLC-γ, among others (Lakshmi et al., 2001). However, few studies have been performed to explore the function of IgD in human T cells.

**FIGURE 1:**
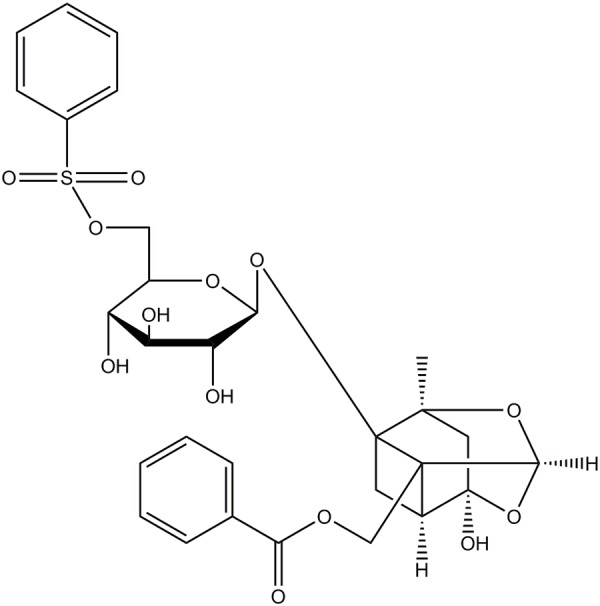
Chemical structures of CP-25 (formula: C29H32O13S; molecular weight: 620).

Recently, our group has identified a new active monomer, paeoniflorin-6′-*O*-benzene sulfonate (code: CP-25; formula: C29H32O13S; molecular weight: 620; patent number in China: ZL201210030616.4) through the structural modification of paeoniflorin (Pae) (Wang et al., 2016; Yang et al., 2016). **Figure 1** shows the chemical construction of CP-25. We reported previously that CP-25 has therapeutic effects on rats with adjuvant-induced arthritis (Chang et al., 2016). We also found that CP-25 could regulate dendritic cell (DC) function and inhibit DC maturation *in vitro* (Li et al., 2015). It can also attenuate the inflammatory response of BAFF-activated CD4^+^ T cells (Jia et al., 2016).

Since CD4^+^ T cell homeostasis is disturbed in autoimmune diseases and IgD is a critical mediator of homeostasis, we hypothesized that CP-25 could rebalance this CD4^+^ T cell homeostasis. We observed that blood levels of IgD were abnormally high in mice with collagen-induced arthritis (data not shown). Here, to simulate the blood environment of RA, we use IgD to stimulate T cells. Therefore, this study aimed to explore the role of IgD in CD4^+^ T cell activation and determine if CP-25 could regulate this process.

We first demonstrated that IgD activates human T cells through IgDR and Lck tyrosine (Tyr^394^) phosphorylation. These data are also the first to demonstrate that CP-25 can inhibit the activation and proliferation of CD4^+^ T cells stimulated by IgD, as well as the production of inflammatory cytokines. We further suggest that this process is probably related to the downregulation of Lck phosphorylation. The results highlight the potential of CP-25 as an ideal and new therapeutic agent for human autoimmune diseases.

## Materials and Methods

### Reagents and Drugs

Human IgD was purchased from Abcam (Cambridge, MA, United States). CP-25 was provided by the Chemistry Lab of the Institute of Clinical Pharmacology of Anhui Medical University with a purity of 98.8% (Hefei, China). CP-25 was dissolved in DMEM. Herbimycin A (HA) was purchased from Millipore (Temecula, CA, United States). A770041 was purchased from Axon Medchem (Groningen, Netherlands). Biotinylated IgD was prepared in our laboratory using a protein biotinylation kit from Pierce Biotechnology (Rockford, IL, United States) according to the manufacturer’s instructions. Human CD4 microbeads were purchased from Miltenyi Biotec (Germany). PE-anti-CD69, PE-anti-CD154, PE-anti-CD62L, PE-anti-IgD, PE-cy5-anti-CD4 monoclonal antibodies (mAbs), APC-Cy7-streptavidin and isotype-matched PE-labeled mouse IgG2a mAbs were purchased from BD Pharmingen (San Diego, CA, United States). The anti-Lck antibody was purchased from Cell Signaling Technology (Danvers, MA, United States).

### Samples

Peripheral blood samples from healthy volunteers, from the First Affiliated Hospital Medical Center, Anhui Medical University, were collected. This study was performed in accordance with the recommendations of the Declaration of Helsinki (2008) and the Ethics Review Committee for the Experimentation of the Institute of Clinical Pharmacology, Anhui Medical University; written informed consent was obtained from all subjects, in accordance with the Declaration of Helsinki. The protocol was approved by the Ethics Review Committee for the Experimentation of the Institute of Clinical Pharmacology, Anhui Medical University (No. 20140192).

### CD4^+^ T Cells Magnetic Separation

Peripheral blood mononuclear cells (PBMCs) were separated by density gradient centrifugation, and CD4^+^ T cells were isolated using magnetic cell separation through positive selection (Miltenyi Biotec, Germany). Labeled T cells were collected after washing with degassed buffer three times. Purity was verified by flow cytometry using PE-cy5 anti-CD4 mAbs. Staining with PE-cy5 anti-CD4 mAb established that isolated CD4^+^ T cells were 96% pure (Supplementary Figure 1), and staining with trypan blue indicated that they were 98% viable.

### T Cell Viability and Proliferation Assay

CD4^+^ T cells were added to 96-well microtiter plates at 2 × 10^5^ cells/well in DMEM with 5% fetal bovine serum (FBS). T cells were cultured in the presence of 3 μg/ml IgD, and incubated for 24 h with the inhibitors HA (1 μmol/l), A770041 (0.5 μmol/l), or CP-25 (10^-7^, 10^-6^, and 10^-5^ mol/l) at 37°C, with 5% CO_2_. For each experiment, the vehicle control group (control) comprised CD4^+^ T cells treated with DMEM and 5% FBS only. T cell viability was assessed using the Cell Counting Kit-8 (WST-8; Dojindo Laboratories, Kumamoto, Japan), and a microplate reader (BioTek Elx-808) was used according to the manufacturer’s protocol. T cell proliferation was assessed using the CFSE Cell Proliferation Kit (BestBio, Shanghai, China) following the protocol of the manufacturer. The working range of CFSE was 0.5–25 μmol/l; however, 4 μmol/l CFSE/10^7^ cells was satisfactory and avoided the toxicity that occasionally occurs with high concentrations of DMSO (used as the solvent for CFSE). After labeling, data were acquired using a flow cytometer (model FC 500; Beckman Coulter Ltd., United States) and data were analyzed with CXP analysis software (Beckman Coulter Ltd., United States, version 2.0).

### Fluorescence-Based Receptor Binding Assay and Scatchard Analysis

The intrinsic binding affinity between the fluorescence-labeled IgD antibody and IgD on IgDR in CD4^+^ T cells was evaluated by fluorescence-based receptor binding assays (Wu et al., 2012; McCall et al., 2014). IgD binding for detection by flow cytometry was performed as follows. Various concentrations of IgD (0.003, 0.01, 0.03, 0.1, 0.3, 1, 3, 10, and 30 μg/ml) were used. Maximal binding of IgD was observed using CD4^+^ T cells in six-well microtiter plates at 1 × 10^6^ cells/well, incubated at 37°C for 2 h in fresh medium (with 0.1% BSA). Cells were then washed three times with PBS at room temperature (RT), and then with buffer appropriate for the assay. IgD binding was detected with a PE-anti-IgD antibody, and isotype-matched PE-labeled mouse IgG2a mAbs were used to assess non-specific binding. The binding of IgD to the anti-IgD antibody was analyzed by flow cytometry. To measure the affinity of the fluorescent IgD ligand, the intensity values corresponding to the maximum fluorescence emission were plotted against total ligand concentrations. Ligand binding properties were evaluated by the fluorescence intensities, assuming that the protein was 100% active, with a stoichiometry of 1:1 (protein:ligand) at saturation. The curves were made linear using Scatchard Plots. The *K*_D_ values for IgD protein were then calculated (Wu et al., 2017).

### Flow Cytometric Analysis

Surface markers of CD4^+^ T cells were evaluated by flow cytometry. CD4^+^ T cells were incubated with PE-anti-CD69 and PE-anti-CD154 mAbs or isotype-matched PE-labeled mouse IgG2a mAbs for 30 min at RT. To determine the expression of IgDR in T cell subsets, human PBMCs were treated with different concentration of IgD and/or CP-25 for 24 h. After incubation, the expression of IgDR was detected using biotinylated IgD followed by APC-Cy7-conjugated streptavidin (Dai et al., 2016; Wu et al., 2016). Cells stained with APC-Cy7 conjugated streptavidin alone were used as an isotype control. After labeling, data were acquired using a flow cytometer and data were analyzed with CXP analysis software. For analysis, the lymphocyte population was gated based on forward and side scatter. Total T cells (CD3^+^), helper T cells (CD3^+^CD4^+^), unactivated T cells (CD4^+^CD62L^+^), and activated T cells (CD4^+^CD69^+^, CD4^+^CD154^+^) were gated. The expression of IgDR on T cells was analyzed by calculating the percentages of CD3^+^IgDR^+^, CD3^+^CD4^+^IgDR^+^, CD4^+^CD62L^+^IgDR^+^, CD4^+^CD69^+^IgDR^+^, and CD4^+^CD154^+^IgDR^+^.

### Western Blotting

CD4^+^ T cells were added to six-well microtiter plates at 5 × 10^6^ cells/well, and treated with different concentrations of IgD and/or CP-25 for 24 h at 37°C and 5% CO_2_. After culture, cells were lysed and centrifuged at 2000 ×*g* for 20 min at 4°C. The supernatants of cultures were collected and maintained at -80°C until use. Protein samples were fractionated by 10% SDS-polyacrylamide gel electrophoresis and transferred to polyvinylidene fluoride microporous membranes (Bio-Rad, Shanghai, China). After blocking with blocking buffer (0.05% Tween 20-PBS with 5% non-fat milk) for 37°C for 2 h, biotinylated IgD was used as the primary antibody for IgDR, overnight at 4°C. Subsequently, cells were treated with HRP-conjugated streptavidin as the secondary antibody (Beyotime Institute of Biotechnology) at 37°C for 2 h. Immunodetection was performed using enhanced chemiluminescence reagent according to the manufacturer’s instructions. Equivalent protein loading and transfer efficiency were verified by β-actin staining. The GSM-3.0 gel graph analyzing system was used to calculate the densitometry of each band.

### Cytokine Assays

The secretion of different cytokines from cultured CD4^+^ T cells was measured by using the Quantibody Human Inflammation Array 1 from Ray Biotech (Norcross, GA, United States). In brief, antibodies against different cytokine [IL-1α, IL-1β, TNF-α, IL-6, IL-10, IL-8, IL-4, INF-γ, IL-13, and monocyte chemotactic protein (MCP)-1] were spotted onto the cytokine array by the manufacturer. CD4^+^ T cells were treated with different concentrations of IgD and/or CP-25 for 24 h in 24-well plates, and incubated at 37°C, 5% CO_2_. Secreted cytokine quantities were measured by adding 100 μl of conditioned medium onto the array for 2 h. Following stringent washes with supplied buffers, detection antibody was added to each well for 1 h at RT and Cy3-equivalent dye-conjugated streptavidin was added for another 1 h at RT to detect bound cytokine. The signal intensity for each spot was determined using an Axon GenePix laser scanner (GenePix 4000B; Molecular Devices, United States) equipped with Cy3 wavelength detection (555 nm excitation, 565 nm emission). The image was analyzed using GenePix Pro 7.0 software, and cytokines were quantified according to the standard curve calibrated from the same array. Outliers were removed and standard curves were then created. Both linear and log regression curves were generated and the curve that gave the best regression line (*r*^2^ closest to 1), was used to determine sample concentrations.

### Statistical Analyses

Statistical analysis was performed by analysis of variance (ANOVA) using SPSS 11.5 software (SPSS, Inc., Chicago, IL, United States). ANOVA was used exclusively for multi group comparisons. Parametric or non-parametric tests were used based on the normality of distribution. Data were presented as the mean ± standard error of the mean unless otherwise indicated. Differences with *P*-values less than 0.05 were considered statistically significant.

## Results

### Binding Affinity between IgD and IgDR

**FIGURE 2:**
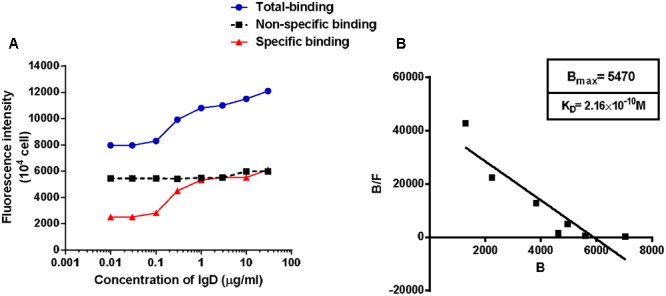
Ligand binding assays on CD4^+^ T cells. Human CD4^+^ T cells expressing immunoglobulin D receptor (IgDR) were incubated with human immunoglobulin D (IgD) for 2 h at 37°C and washed three times. Cells with bound fluorescent ligands were determined by flow cytometry. Cells (1 × 10^4^) were collected for each sample and the ligand binding properties were evaluated by assessing the fluorescence intensities of 1 × 10^4^ cells. IgD binding was detected with PE-anti-IgD antibody, and isotype-matched PE-labeled mouse IgG2a monoclonal antibodies (mAbs) were used to calculate non-specific binding. Values for non-specific binding, determined using the isotype control, were subtracted. Data are plotted as Scatchard analyses and are representative of several independent experiments, *n* = 5. **(A)** Total, non-specific, and specific binding vs. IgD concentrations were plotted. **(B)** The Scatchard line was transformed from the specific binding values. *B*_max_ and *K*_D_ values were calculated by Scatchard plot analysis.

To determine whether the observed binding of IgD to IgDR on CD4^+^ T cells occurred with the characteristics of receptor–ligand interaction, experiments were designed to demonstrate receptor saturation and binding affinity. CD4^+^ T cells were separated from healthy donors using magnetic beads. The affinity of human IgD was detected using a PE-conjugated anti-IgD antibody. Binding of IgD to IgDR, expressed on T cells, was also determined using IgD as the ligand. Binding between IgD and IgDR was clearly concentration-dependent (**Figure 2A**). We estimated the IgD binding affinity by performing a fluorescence-based receptor binding assay. Total, non-specific, and specific binding vs. IgD concentrations were plotted as shown in **Figure 2A**. The Scatchard line transformed from the specific binding data was plotted as shown in **Figure 2B**. The *B*_max_ and *K*_D_ values were calculated by Scatchard plot analysis. The *K*_D_ value was 2.16 × 10^-10^ mol/l, which represents high-affinity, and the *B*_max_ value was 5470 arbitrary units/10^4^ cells (**Figure 2B**).

### Effect of IgD on T Cell Proliferation and Activation

**FIGURE 3:**
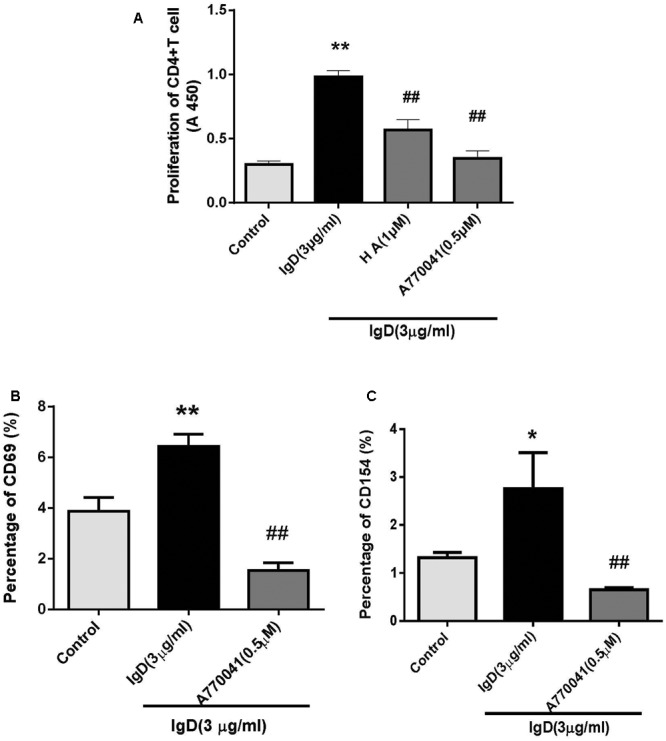
Effect of protein tyrosine kinase (PTK) inhibitors on the stimulatory effect of immunoglobulin D (IgD) in CD4^+^ T cells. Human CD4^+^ T cells were cultured with IgD (3 μg/ml) and inhibitors herbimycin A (HA; 1 μmol/l) or A770041 (0.5 μmol/l) for 24 h. Trypan blue-labeled CD4^+^ T cells in each group indicated that cells were 98% viable. **(A)** Proliferation of CD4^+^ T cells, as detected by CCK-8. Flow cytometry was used to analyze the proportions of CD69^+^ **(B)** and CD154^+^ **(C)** in CD4^+^ T cell populations. ^∗^*P* < 0.05, ^∗∗^*P* < 0.01 vs. control; ^#^*P* < 0.05, ^##^*P* < 0.01 vs. IgD (3 μg/ml) group.

To determine if IgD activates CD4^+^ T cells via PTK signaling pathways and the phosphorylation of Lck, we used the PTK inhibitor HA and the Lck inhibitor A770041. Subsequently, we first investigated the effects of IgD on T cell proliferation. T cells were incubated for 18 h with HA and for 24 h with A770041 separately before culturing in the presence of 3 μg/ml IgD. As shown in **Figure 3A**, IgD (3 μg/ml) significantly promoted the proliferation of CD4^+^ T cells, whereas HA (1 μmol/l) or A770041 (0.5 μmol/l) treatment markedly inhibited IgD-induced proliferation. Next, the expression of CD69 and CD154 on T cells treated with IgD for 24 h was analyzed by flow cytometry (**Figures 3B,C**). Significant increases in the proportions of CD69^+^ and CD154^+^ subsets in CD4^+^ T cell populations were caused by IgD (3 μg/ml), whereas A770041(0.5 μmol/l) significantly suppressed this effect.

### IgD Upregulates IgDR Expression and Lck Phosphorylation in CD4^+^ T Cells

**FIGURE 4:**
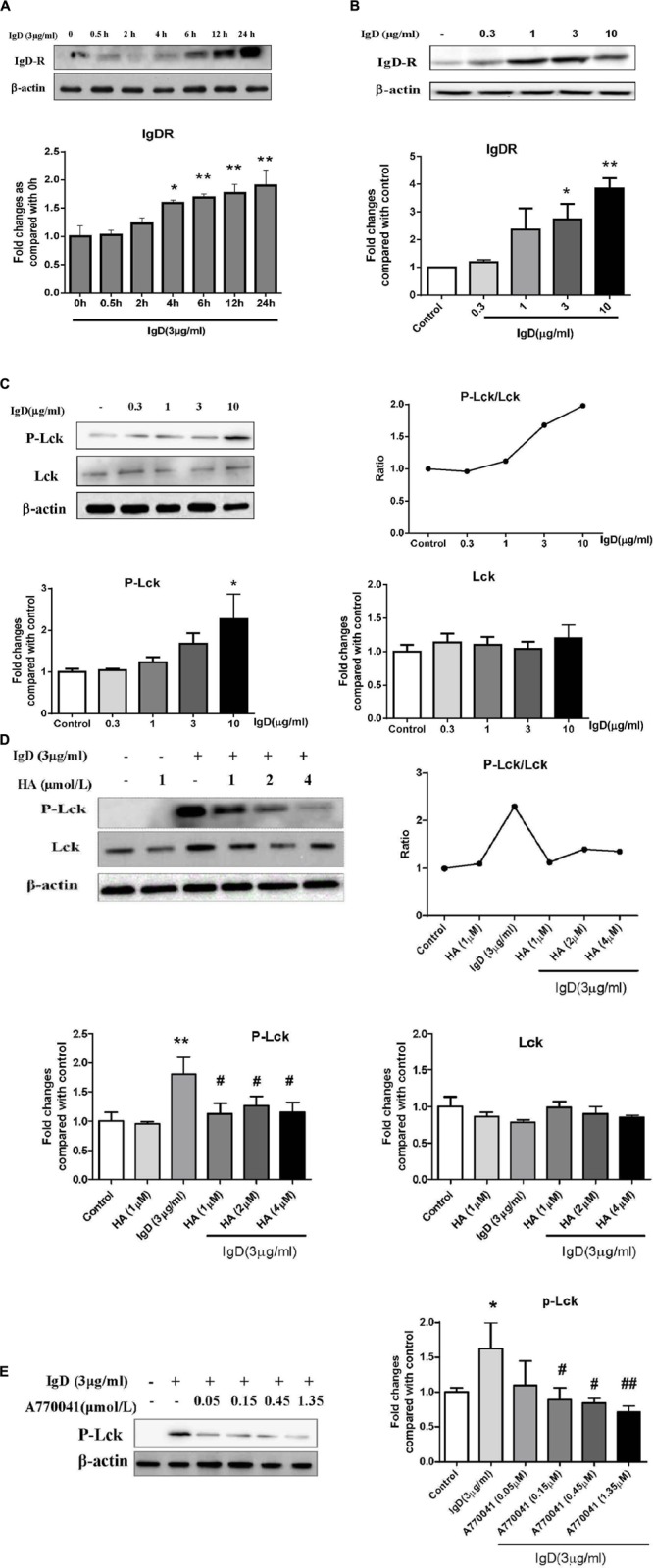
Effect of immunoglobulin D (IgD) and inhibitors on the expression of immunoglobulin D receptor (IgDR), Lck, and P-Lck in human CD4^+^ T cells. Data are expressed as the mean ± standard error of the mean (*n* = 3). **(A)** Western blot analysis of IgDR expression in CD4^+^ T cells stimulated with IgD at different times (0–24 h); ^∗^*P* < 0.05, ^∗∗^*P* < 0.01 vs. 0 h. Western blot analysis of IgDR **(B)**, Lck **(C)**, and P-Lck **(C)** expression in CD4^+^ T cells stimulated with IgD at different concentrations (1∼10 μg/ml); ^∗^*P* < 0.05, ^∗∗^*P* < 0.05 vs. control. Western blot analysis of Lck and P-Lck expression in CD4^+^ T cells stimulated with IgD (3 μg/ml) after pretreatment with herbimycin A **(D)** and A770041 **(E)**; ^∗^*P* < 0.05, ^∗∗^*P* < 0.01 vs. control, ^#^*P* < 0.05, ^##^*P* < 0.01 vs. IgD (3 μg/ml) group.

As the amount of IgDR on activated T cells was enhanced by IgD, we further examined whether this treatment could enhance the expression of IgDR itself in CD4^+^ T cells by performing western blots. CD4^+^ T cells were stimulated by IgD (3 μg/ml) for 0.5, 2, 4, 6, 12, and 24 h. As expected, the protein expression of IgDR (approximately 70 kDa) increased after a 12 h-co-culture with IgD. For the next experiment, we chose 24 h as an optimal time for stimulation (**Figure 4A**). Furthermore, the expression of IgDR was increased in response to treatment with IgD in a concentration-dependent manner (**Figure 4B**, *P* < 0.05). The expression could also be increased by treatment with phytohemagglutinin (PHA, 4 μg/ml; Sigma, St. Louis, MO, United States). Lck is critically involved in the initial phase of T cell signal transduction. We thus examined whether treating CD4^+^ T cells with IgD could result in activation of Lck (Tyr^394^) tyrosine phosphorylation. As shown in **Figure 4C**, sIgD stimulation resulted in enhanced Lck phosphorylation compared to that in the medium control sample. The expression of Lck was not changed. We used the inhibitor HA and Lck to separately investigate the effects IgD on inhibition of PTK signaling and Lck phosphorylation. For inhibition experiments, T cells were incubated for 18 h with HA and for 24 h with A770041 separately before administering 3 μg/ml IgD. As shown in **Figures 4D,E**, HA (1, 2, and 4 μmol/l) or A770041 (0.15, 0.45, and 1.35 μmol/l) inhibited IgD-induced Lck phosphorylation.

### CP-25 Inhibits IgD-Induced PBMC and CD4^+^ T Cell Proliferation

**FIGURE 5:**
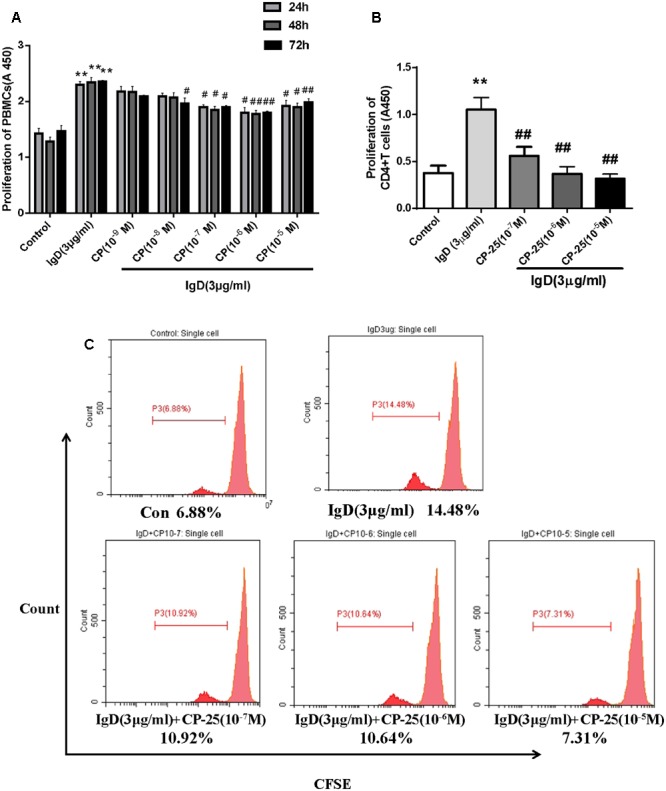
Effect of CP-25 on proliferation of human peripheral blood mononuclear cells (PBMCs) and CD4^+^ T cells stimulated with immunoglobulin D (IgD) *in vitro.* CD4^+^ T cells in each group were 98% viable, based on trypan blue staining. PBMCs were cultured with different concentrations of CP-25 (10^-9^∼10^-5^ mol/l) combined with IgD (3 μg/ml) for 24, 48, and 72 h, and the viability of PBMCs was detected by CCK-8 **(A)**. Purified CD4^+^ T cells were cultured with IgD alone or CP-25 in addition. The viability and proliferation of T cells were separately detected by CCK-8 **(B)** and CFSE analysis **(C)**. Data are expressed as the mean ± standard error of the mean (*n* = 3). ^∗^*P* < 0.05, ^∗∗^*P* < 0.01 vs. control; ^#^*P* < 0.05, ^##^*P* < 0.01 vs. IgD (3 μg/ml) group.

PBMCs were cultured with different concentrations of CP-25 (10^-9^∼10^-5^ mol/l) combined with IgD (3 μg/ml) for 24, 48, and 72 h. IgD significantly promoted proliferation in PBMCs, whereas CP-25 (10^-7^∼10^-5^ mol/l) treatment markedly inhibited IgD-induced proliferation after 24 h of culture (**Figure 5A**, *P* < 0.05). As such, CP-25 had no significant effect on PBMC proliferation (data not shown). Next, we investigated the effect of CP-25 on IgD-induced CD4^+^ T cell proliferation. Of note, CP-25 (10^-7^∼10^-5^ mol/l) treatment markedly inhibited IgD-induced proliferation after 24 h of culture (**Figure 5B**, *P* < 0.01). CFSE dye irreversibly binds cytoplasmic proteins and is evenly distributed between daughter cells during cell division. Flow cytometry revealed a cell population with lower fluorescence intensity compared to that in initial cells (**Figure 5C**). Consistently, IgD (3 μg/ml) stimulated the proliferation of CD4^+^ T cells, as compared to that in the control group. The suppression of proliferation was pronounced after CP-25 (10^-7^∼10^-5^ mol/l) treatment, as characterized by a deceleration of cell division.

### CP-25 Suppresses IgD-Enhanced T Cell Subset Activation

**FIGURE 6:**
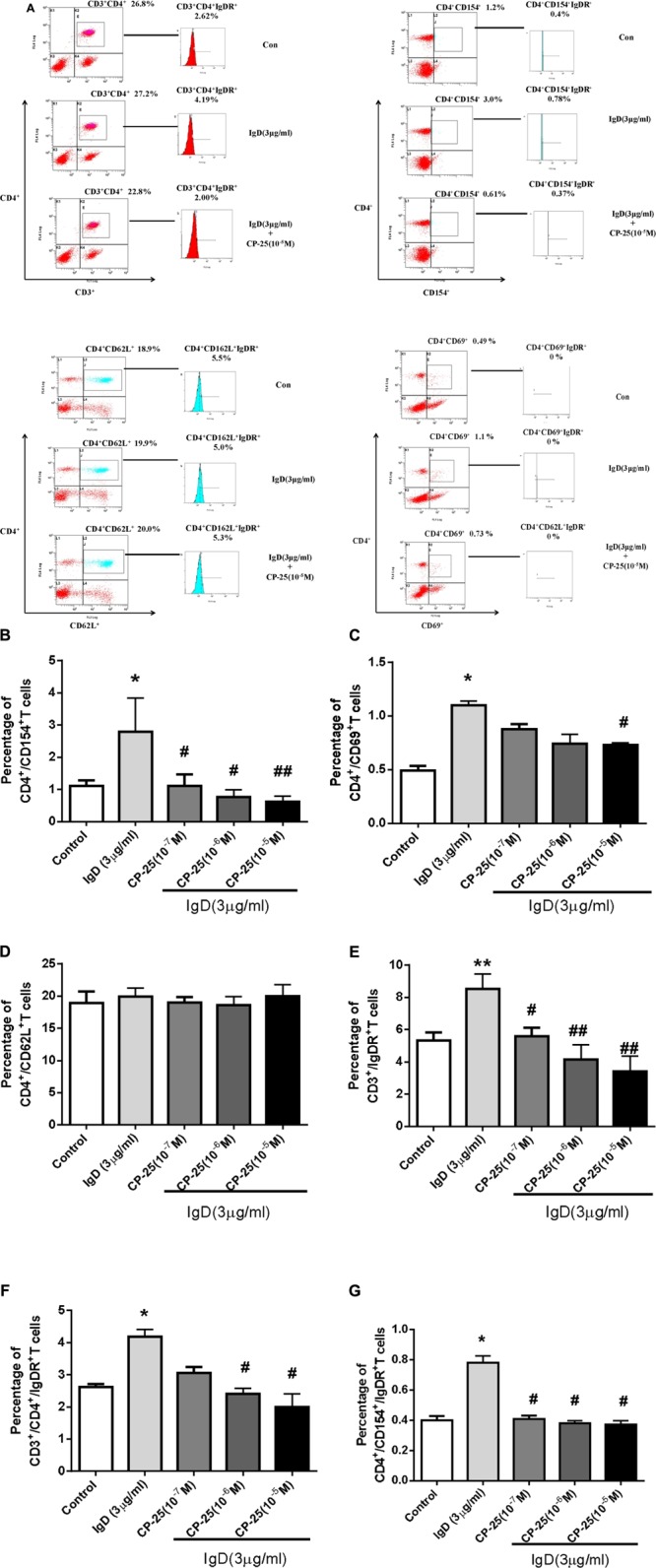
Effect of CP-25 on the proportions of T cell subsets and the expression of immunoglobulin D receptor (IgDR) in human peripheral blood mononuclear cells (PBMCs) induced by immunoglobulin D (IgD). Human PBMCs were cultured with different concentrations of CP-25 (10^-9^∼10^-5^ mol/l) combined with IgD (3 μg/ml) for 24 h. Flow cytometry was used to analyze the effects of CP-25. After incubation, the expression of IgDR was detected using biotinylated IgD followed by APC-Cy7-conjugated streptavidin. Cells stained with APC-Cy7 conjugated streptavidin alone were used as an isotype control. **(A)** Representative flow cytometry dot plot from each group. Bar graph illustrating the proportion of CD4^+^/CD154^+^ **(B)**, CD4^+^/CD69^+^ **(C)**, CD4^+^/CD62L^+^ **(D)**, CD3^+^/IgDR^+^ **(E)**, CD3^+^/CD4^+^/IgDR^+^ **(F)**, and CD4^+^/CD154^+^/IgDR^+^ **(G)** T cells. Data are expressed as the mean ± standard error of the mean (*n* = 5). ^∗^*P* < 0.05, ^∗∗^*P* < 0.01 vs. control group; ^#^*P* < 0.05, ^##^*P* < 0.01 vs. IgD group.

Next, the effects of CP-25 on T cell activation were analyzed (**Figure 6**). Results showed that the proportions of activated T cells (CD4^+^CD69^+^, CD4^+^CD154^+^; *P* < 0.05) increased after co-culturing with IgD (3 μg/ml) for 24 h. IgD had no significant effect on total helper T cells (CD3^+^CD4^+^) or unactivated T cells (CD4^+^CD62L^+^). CP-25 (10^-7^∼10^-5^ mol/l) significantly decreased the proportion of CD4^+^CD154^+^ induced by IgD (**Figure 6B**, *P* < 0.05). CP-25 (10^-5^ mol/l) significantly decreased the proportion of CD4^+^CD69^+^ cells induced by IgD (**Figure 6C**, *P* < 0.05). Accordingly, CP-25 had no significant effect on each T cell subset (Supplementary Figure 2).

### CP-25 Decreases the IgD-Mediated Expression of IgDR in T Cell Subsets

The expression of IgDR on T cell subsets was analyzed after treatment with different concentrations of IgD and/or CP-25 for 24 h. A significant increase in the proportions of CD3^+^IgDR^+^, CD3^+^CD4^+^IgDR^+^, and CD4^+^ CD154^+^IgDR^+^ T cells was observed with IgD (3 μg/ml; **Figure 6**, *P* < 0.05) in PBMC. IgD had no significant effect on the proportions of CD4^+^CD62L^+^IgDR^+^ T cells. CP-25 (10^-7^∼10^-5^ mol/l) significantly decreased the proportion of CD3^+^IgDR^+^ and CD4^+^CD154^+^IgDR^+^ T cells induced by IgD after 24 h of culture (**Figures 6E,G**, *P* < 0.05). Further, CP-25 (10^-6^∼10^-5^ mol/l) significantly decreased the percentage of CD3^+^CD4^+^IgDR^+^ T cells induced by IgD (3 μg/ml) after 24 h of culture (**Figure 6F**, *P* < 0.05). The percentage of CD4^+^CD69^+^IgDR^+^ T cells was too low to be detected.

### CP-25 Decreases the Production of Inflammatory Cytokines Secreted by IgD-Activated T Cells

**FIGURE 7:**
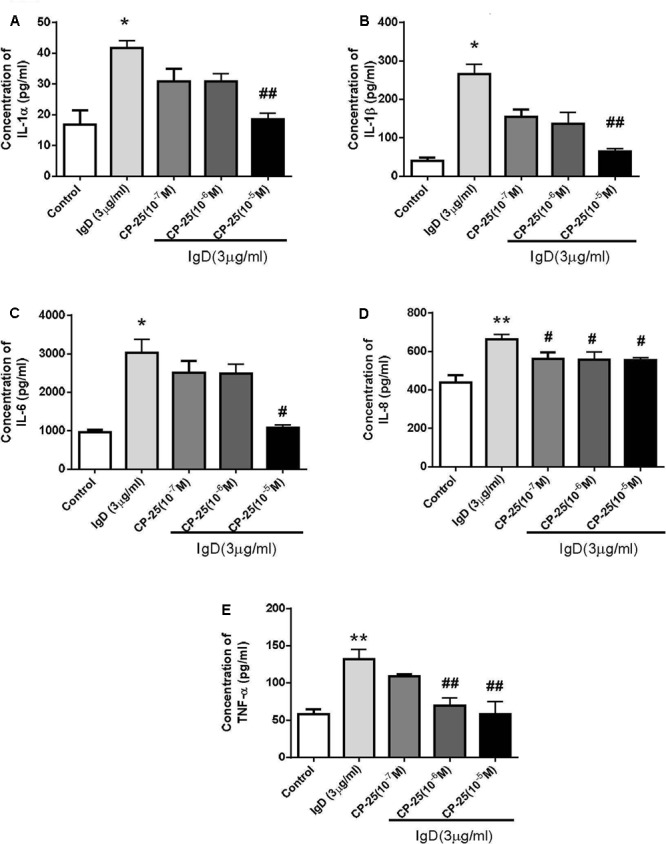
Variation in inflammatory cytokine secretion by human CD4^+^ T cells stimulated by immunoglobulin D (IgD) and the effect of CP-25. Cell culture supernatants were collected after treated with IgD (3 μg/ml) and CP-25 (10^-7^∼10^-5^ mol/l) for 24 h. The glass slide was scanned using a microarray scanner, and levels of IL-1α **(A)**, IL-1β **(B)**, IL-6 **(C)**, IL-8 **(D)**, and TNF-α **(E)** were measured. Data are expressed as the mean ± standard error of the mean (*n* = 4). ^∗^*P* < 0.05, ^∗∗^*P* < 0.01 vs. control; ^#^*P* < 0.05, ^##^*P* < 0.01 vs. IgD group.

To examine whether CP-25 affects inflammatory cytokine secretion by IgD-activated T cells, inflammatory cytokines were analyzed by the Quantibody Human Inflammation Array. Compared to those in the control group, the concentrations of IL-1α, IL-1β, IL-6, IL-8, and TNF-α were increased significantly with the treatment of IgD (3 μg/ml; *P* < 0.05; **Figure 7**). There were no obvious differences in the concentrations of IL-10, IL-4, INF-γ, IL-13, and MCP-1. As shown in **Figure 7**, CP-25 (10^-5^ mol/l) significantly decreased the production of IL-1α, IL-1β, IL-6 induced by IgD after 24 h of culture; moreover, CP-25 (10^-6^∼10^-5^ mol/l) decreased the production of TNF-α and CP-25 (10^-7^∼10^-5^ mol/l) decreased the production of IL-8.

### CP-25 Downregulates IgD-Induced IgDR Expression and Lck Phosphorylation

**FIGURE 8:**
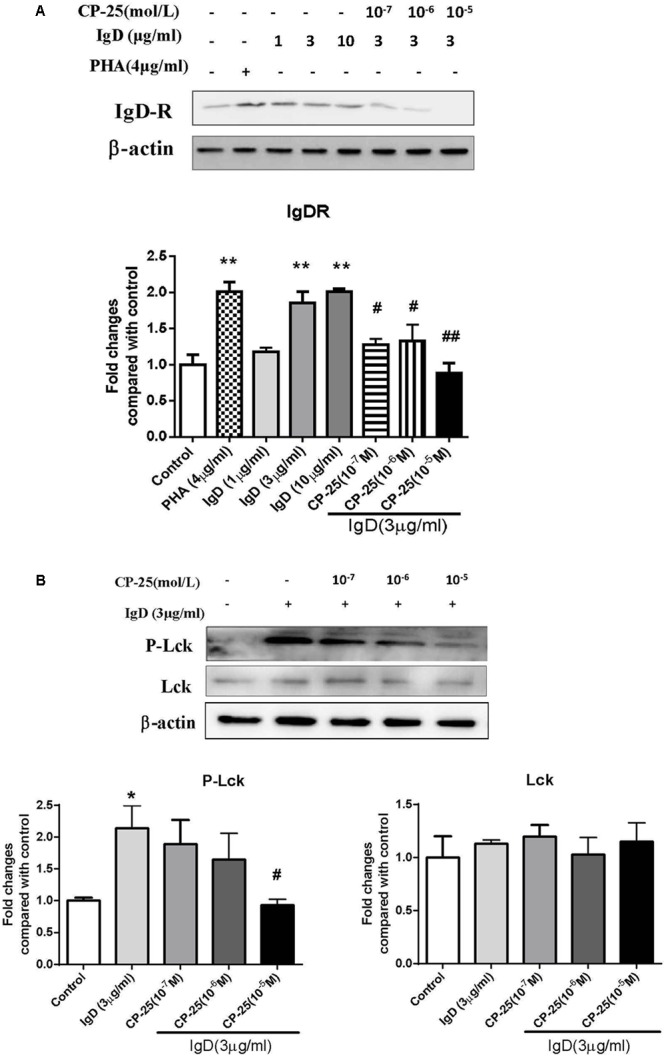
Effects of CP-25 on the expression of immunoglobulin D receptor (IgDR) and Lck in human CD4^+^ T cells stimulated with immunoglobulin D (IgD). CD4^+^ cells were treated with IgD (3 μg/ml) and CP-25 (10^-7^∼10^-5^ mol/l) for 24 h and lysed. **(A)** Western blot analysis of IgDR expression in CD4^+^ T cells. **(B)** Western blot analysis of Lck and P-Lck expression in CD4^+^ T cells. Data are expressed as the mean ± standard error of the mean (*n* = 3). ^∗^*P* < 0.05, ^∗∗^*P* < 0.01 vs. control; ^#^*P* < 0.05, ^##^*P* < 0.01 vs. IgD (3 μg/ml) group.

We found that CP-25 can inhibit the activation of CD4^+^ T cells induced by IgD, and further examined whether CP-25 could rebalance CD4^+^ T cell homeostasis by downregulating total IgDR protein expression and Lck phosphorylation (Tyr^394^). As expected, CP-25 (10^-7^∼10^-5^ mol/l) downregulated the total protein expression of IgDR (**Figure 8A**), and CP-25 (10^-5^ mol/l) significantly downregulated IgD-induced Lck phosphorylation to control levels (**Figure 8B**).

## Discussion

IgD plays a key role in innate immunity and inflammation (Chen and Cerutti, 2010). Whereas the function of IgM, IgG, IgA, and IgE is relatively well-known, the function of IgD has remained obscure for a significant amount of time. Low concentrations of IgD are secreted under physiological conditions (Rowe et al., 1973; Walsh and Kay, 1986), whereas increased IgD can occur in atypical situations such as IgD myeloma, skin allergy, hyper IgE and hyper IgD syndromes, and autoimmune disorders such as RA and SLE (Rostenberg and Penaloza, 1978; Drenth et al., 1996; Sechet et al., 2003). However, high expression of sIgD was found to be associated with high levels of protein-like sediments and cell necrosis in the kidney, spleen, and liver of transgenic mice (Wang et al., 2012); further, recent studies have suggested that IgD might play an important role in autoimmune diseases. The function of IgD has remained elusive since its discovery, despite numerous attempts to define its biological purpose. Surprisingly, the existence of an Fc receptor specific for IgD (FcR or IgDR) is still not entirely clear. Its existence on T cells has been suggested (Coico et al., 1985). *In vivo* and *in vitro* studies have suggested that a putative IgDR on T cells might play a role in the regulation of IgD (Drenth et al., 1996; Lakshmi et al., 2001; Sechet et al., 2003). Recently, our group reported further evidence for the existence of an IgDR on B cells (Wu et al., 2016), the human Burkitt lymphoma B cell line Daudi (Dai et al., 2016), and human fibroblast-like synoviocytes (FLSs; Wu et al., 2017).

T cell activation usually occurs with increased expression of CD69 and CD154 (CD40L) (Canavan et al., 2012). We reported that the proportions of activated T cells (CD4^+^CD69^+^, CD4^+^CD154^+^) increases after co-culture with IgD both in RA patients and in healthy controls. In particular, IgD was shown to induce a rapid increase in CD4^+^IgDR^+^ T cells in RA patients (Wu et al., 2016). CD4^+^ T cells, which are critical elements of vertebrate adaptive immune responses, are produced in the thymus and migrate to the periphery where they encounter antigens. Whereas aberrant control of Th immune response is implicated in the progression of autoimmune diseases such as RA, SLE, ulcerative colitis (Yamada et al., 2002; Goronzy et al., 2005). Our group has proposed that CD4^+^ T cells might be key effectors in RA patients. Excessive IgD probably causes the activation of CD4^+^ T cells, resulting in the abnormal proliferation of PBMCs and increased secretion of inflammatory cytokines.

To clearly illustrate the mechanism associated with IgD on CD4^+^ T cells, we determined the effect of IgD on CD4^+^ T cells isolated from humans. We found that IgD binds IgDR on T cells with high affinity. A significant increase in the proportion of CD69^+^ and CD154^+^ cells in CD4^+^ T populations was caused by IgD. Simultaneously, the expression of IgDR on activated T cells (CD4^+^/CD154^+^) was elevated after IgD stimulation. Our data suggested that IgD promotes the activation of T cells by stimulating the expression of IgDR. In this study, IgD significantly enhanced the proliferation of CD4^+^ T cells and increased levels of inflammatory cytokines including IL-α, IL-1β, IL-6, IL-8, and TNF-α secreted by CD4^+^ T cells, which was consistent with previous reports on PBMCs from RA patients (Chen et al., 2009; Wu et al., 2016). IL-1, IL-6, and TNF-α are key inflammatory cytokines that play a critical role in T cell activation, tissue destruction, and joint inflammation in RA (Kloesch et al., 2013; Jin et al., 2016). Our results demonstrated that IL-1, IL-6, and TNF-α are released from activated T cells; in addition, to date, IgD has been considered an important participant in the pathophysiology of RA.

Further, functional analysis of IgDR and its signaling pathway were performed in this study to provide a clearer explanation of the molecular mechanisms that regulate T cell activation. Reports focusing on IgDR-related signaling were previously limited. In mice, crosslinking of IgDR might induce signal transduction through the activation of PTK pathways (Amin et al., 1993); total protein expression of IgDR and phosphorylation of Lck were previously shown to be enhanced by IgD in mouse T cells (Lakshmi et al., 2001). Furthermore, the tyrosine kinase Lck plays a crucial role in the maturation of lymphocytes in the thymus and in mature T cell activation and proliferation (Gascoigne and Palmer, 2011; Barrera-Vargas et al., 2014; Klammt et al., 2015; Vahedi et al., 2015). Lck hyperactivation leads to an overall enhanced TCR downstream signaling, including JNK activation TCR signaling (Li et al., 2014). Dysregulation of the expression and activity of Lck was described in lymphocytes from SLE patients (Barrera-Vargas et al., 2014). Inhibitors of Lck are suggested to be efficacious for the treatment of inflammatory-immune diseases including RA, MS, inflammatory bowel diseases, type 1 diabetes, SLE, and psoriasis (Stachlewitz et al., 2005). In this study, we found that the total protein expression of IgDR and the activation of Lck (Tyr^394^) tyrosine phosphorylation were enhanced by IgD in CD4^+^ T cells. However, the expression of Lck was not changed. A previous study showed that HA, which is a tyrosine kinase inhibitor, can block PTK signaling and significantly inhibit the induction of IgDR in murine cells (Amin et al., 1993; Swenson et al., 1993). We used the inhibitors HA and A770041 to confirm whether Lck is the key molecular mediator of IgD-induced T cell activation. A770041 is a selective inhibitor of Lck (Stachlewitz et al., 2005; Burchat et al., 2006; Duan et al., 2014). Both HA and A770041 could significantly inhibit the phosphorylation of Lck after IgD stimulation. Compared to that in the control, the activating effect on proliferation caused by IgD was blocked by HA or A770041 in CD4^+^ T cells. A770041 could significantly attenuate the IgD-induced proportion of CD69^+^ and CD154^+^ cells in CD4^+^ T cells. This finding might demonstrate that IgD can upregulate the expression of IgDR, activate CD4^+^ T cells, and promote the proliferation and secretion of inflammatory cytokines in CD4^+^ T cells by enhancing the phosphorylation of Lck (Tyr^394^). Our results support the hypothesis that IgD plays an important role in the activation of T cells in pathogenesis of RA.

CP-25, a novel ester derivative of Pae, was synthetized by our group. The protective effects of this compound during adjuvant-induced arthritis have been reported recently (Chang et al., 2016). *In vitro* studies have illustrated that CP-25 inhibits the growth and cytokine secretion ability of FLSs in a rat model of RA (Jia et al., 2016). Its therapeutic effect was related to the regulation of T/B cells and FLSs. Thus, we assume that CP-25 can suppress the abnormal activation of T cells induced by IgD, and that the mechanism through which CP-25 inhibits the activation of T cells is related to the expression of IgDR and Lck.

In this study, data showed that CP-25 significantly decreases proliferation and inflammatory cytokine secretion in IgD-induced CD4^+^ T cells *in vitro*. CP-25 reduced the secretion of cytokines to a similar level to that observed in the unstimulated group. Flow cytometry results showed that CP-25 decreases the proportion of IgD-induced activated T cells (CD4^+^/CD154^+^, CD4^+^/CD69^+^). Simultaneously, CP-25 could also reduce the expression of IgDR on activated T cells (CD4^+^/CD154^+^), of which the level was otherwise increased due to IgD, whereas CP-25 had no significant effect on total and immature T cells. Moreover, CP-25 could significantly decrease the IgD-induced expression of IgDR and the phosphorylation of Lck in CD4^+^ T cells. We observed that the level of IgD in blood was abnormally high in mice with collagen-induced arthritis (data not shown). Here, we used IgD to stimulate T cells to simulate the blood environment of RA patients. These results indicated that IgD promotes the activation of CD4^+^ T cells, and that CP-25 could selectively act on active T cells, decreasing the levels of active T cells to control levels, by regulating the phosphorylation of Lck. We observed that CP-25 could cross the cell membrane into the cytoplasm (data not shown). As such, one possible mechanism for this is that CP-25 inhibits the phosphorylation of Lck, inhibits the activation of T cells, down regulates the expression of IgDR, and then decreases the binding of IgD to the overexpressed IgDR. This mechanism might be related to the “soft regulation of inflammatory immune responses (SRIIR)” overexpressing IgDR on T cells. SRIIR, which was proposed by our group recently (Wei, 2016), is a new concept for the discovery and development of new drugs for the treatment of inflammatory immune responses related diseases. It refers to drugs that do not completely suppress cell function, as well as gene and protein expression or activity, but regulate selectively abnormal activity to achieve physiological levels, restore dynamic balance, and play a role in the treatment and reduction of adverse reactions. SRIIR provides a crucial direction for the development of innovative drugs. Our results indicated that CP-25 probably is a SRIIR drug, which could mildly decrease the production of activated T cells and reduce the excessive secretion of inflammatory cytokines to physiological levels.

Taking these data together, our study showed that IgD takes part in activating CD4^+^ T cells via the IgDR–Lck axis, which was consistent with our idea that the abnormally high level of IgD released by B cells plays an important role in RA. CP-25 can inhibit the IgD-stimulated activation and proliferation of CD4^+^ T cells, as well as the production of inflammatory cytokines, and this process is probably related to the downregulation of Lck phosphorylation. The results highlight the potential for CP-25 to be a novel and ideal agent to treat RA and other autoimmune diseases.

## Author Contributions

Y-jW designed and performed experiments, and wrote the manuscript. H-sC participated in the design of the study, performed the experiments and statistical analysis. W-sC and JD performed experiments. X-jD and XD carried out the flow cytometry assays. QH helped to revise the manuscript. WW conceived of the study and revised the manuscript. All authors read and approved the final manuscript.

## Conflict of Interest Statement

The authors declare that the research was conducted in the absence of any commercial or financial relationships that could be construed as a potential conflict of interest.

## Abbreviations

CP-25: paeoniflorin-6′-*O*-benzene sulfonate
FLSs: fibroblast-like synoviocytes
HA: herbimycin A
IgD: immunoglobulin D
IgDR: immunoglobulin D receptor
mIgD: membrane IgD
MS: multiple sclerosis
PBMC: peripheral blood mononuclear cell
PHA: phytohemagglutinin
PTK: protein tyrosine kinase
RA: rheumatoid arthritis
sIgD: secreted IgD
SLE: systemic lupus erythematosus
SRIIR: soft regulation of inflammatory immune responses
UC: ulcerative colitis
