# Supplementary material for: CP-25 Attenuates the Activation of CD4+ T Cells Stimulated with Immunoglobulin D in Human
Source: Front Pharmacol. 2018 Jan 23;9:4. doi: 10.3389/fphar.2018.00004 (PMC5787084; doi:10.3389/fphar.2018.00004)
Supplement: Supplementary file 1 [file Image_1.PDF]

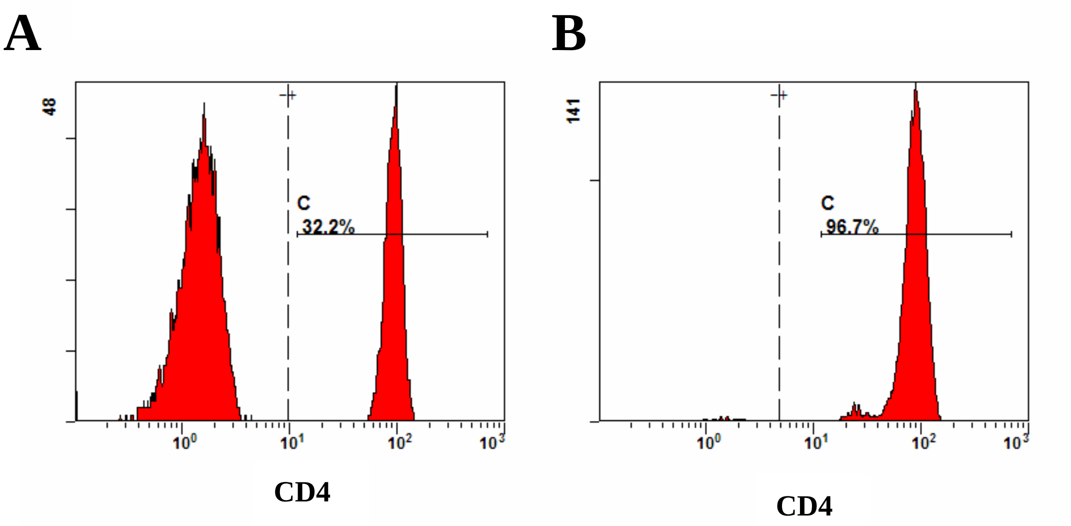

Supplyment Fig 1 Identification of CD4^{+} cells purity in human PBMCs. Flow cytometry analysis was used to identify the purity of CD4^{+} cells.(A) The purity of CD4^{+} cells before separated by magnetic beads. (B) The purity of CD4^{+} cells after separated by magnetic beads.

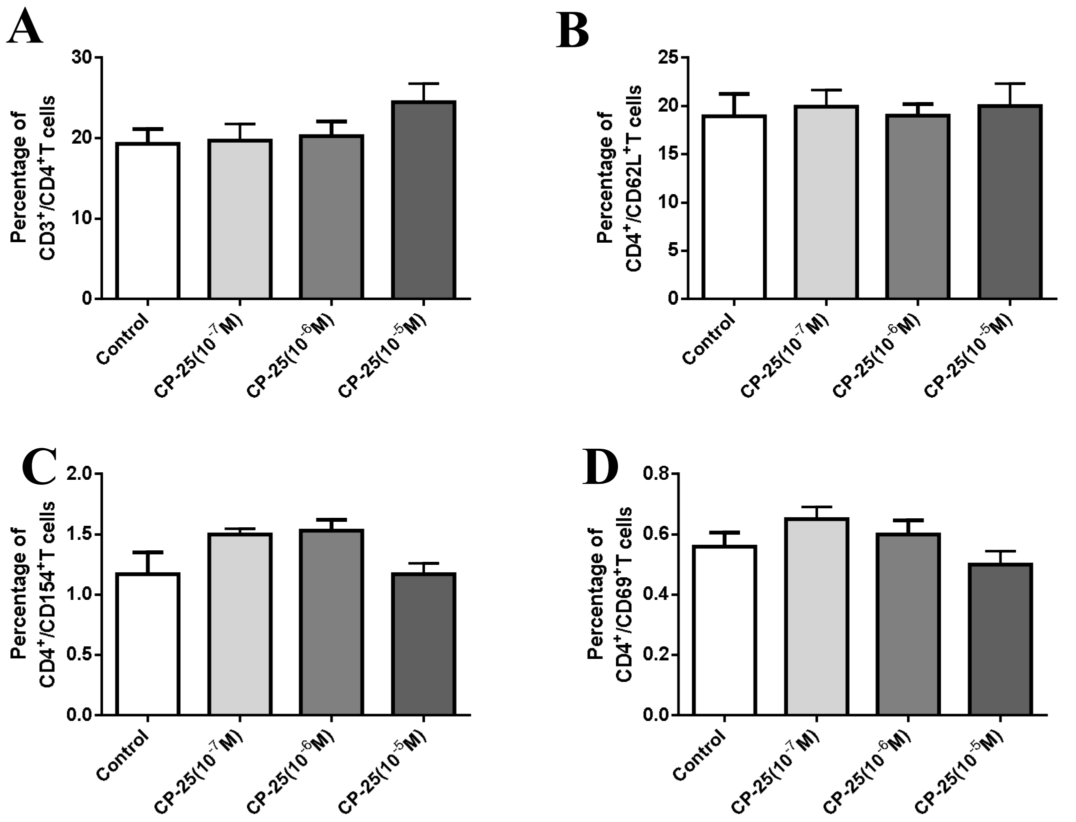

Supplyment Fig 2 Effect of CP-25 on the expression of T cells subsets in human PBMCs. Flow cytometry analysis was used to analyze the effects of CP-25(10^{-7}~10^{-5} mol/L) on the percentages of $CD3^+/CD4^+$ (A), $CD4^+/CD62L^+$ (B), $CD4^+/CD154^+$ (C) and $CD4^+/CD69^+$ (D)T cells. Data are expressed as the mean ± standard error of the mean (n=3).
